# (*R*)-(−)-*N*-Isovalerylcamphorsultam

**DOI:** 10.1107/S1600536809035120

**Published:** 2009-09-09

**Authors:** Wei Zhou, Shijie Zhang, Weixiao Hu

**Affiliations:** a College of Pharmaceutical Science, Zhejiang University of Technology, Hangzhou 310014, People’s Republic of China

## Abstract

The title compound, C_15_H_25_NO_3_S, was prepared from (*R*)-(−)camphorsultam and isovaleryl chloride. The asymetric unit contains two independent mol­ecules with slightly different conformations. In the crystal, weak inter­molecular C—H⋯O hydrogen bonds link mol­ecules into two independent hydrogen-bonded chains propagating along the *a* and *b* axes.

## Related literature

The title compound is used to obtain a key intermediate in the synthesis of the new renin inhibitor Aliskiren. For the properties of Aliskiren, see Mariano *et al.* (2008 ▶).
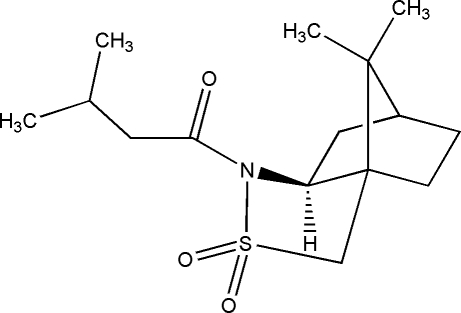


## Experimental

### 

#### Crystal data

C_15_H_25_NO_3_S
*M* *_r_* = 299.42
Tetragonal, 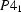

*a* = 7.9038 (7) Å
*c* = 50.228 (8) Å
*V* = 3137.8 (7) Å^3^
*Z* = 8
Mo *K*α radiation
μ = 0.21 mm^−1^
*T* = 293 K
0.30 × 0.30 × 0.20 mm

#### Data collection

Bruker SMART CCD area-detector diffractometer
Absorption correction: multi-scan (*SADABS*; Sheldrick, 1996 ▶) *T*_min_ = 0.939, *T*_max_ = 0.959
16320 measured reflections
6416 independent reflections
5184 reflections with *I* > 2σ(*I*)
*R*_int_ = 0.035

#### Refinement

*R*[*F* ^2^ > 2σ(*F* ^2^)] = 0.045
*wR*(*F* ^2^) = 0.109
*S* = 1.04
6416 reflections
370 parameters
1 restraint
H-atom parameters constrained
Δρ_max_ = 0.38 e Å^−3^
Δρ_min_ = −0.19 e Å^−3^
Absolute structure: Flack (1983 ▶), 2779 Friedel pairs
Flack parameter: 0.03 (6)

### 

Data collection: *SMART* (Bruker, 1997 ▶); cell refinement: *SAINT* (Bruker, 1997 ▶); data reduction: *XCAD4* (Harms & Wocadlo, 1995 ▶); program(s) used to solve structure: *SHELXS97* (Sheldrick, 2008 ▶); program(s) used to refine structure: *SHELXL97* (Sheldrick, 2008 ▶); molecular graphics: *ORTEP-3 for Windows* (Farrugia, 1997 ▶); software used to prepare material for publication: *SHELXL97*.

## Supplementary Material

Crystal structure: contains datablocks global, I. DOI: 10.1107/S1600536809035120/cv2605sup1.cif

Structure factors: contains datablocks I. DOI: 10.1107/S1600536809035120/cv2605Isup2.hkl

Additional supplementary materials:  crystallographic information; 3D view; checkCIF report

## Figures and Tables

**Table 1:** Hydrogen-bond geometry (Å, °)

*D*—H⋯*A*	*D*—H	H⋯*A*	*D*⋯*A*	*D*—H⋯*A*
C4—H4*A*⋯O3^i^	0.97	2.45	3.387 (3)	162
C15′—H15*F*⋯O3′^ii^	0.96	2.49	3.450 (5)	173

Symmetry codes: (i) 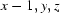; (ii) 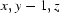.

**Fig. 1.**
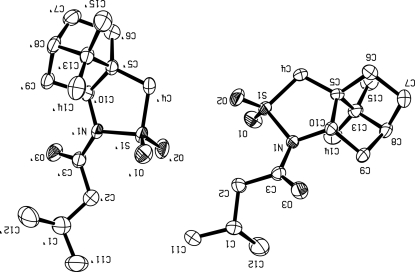
The content of asymmetric part of (1) showing the atomic numbering and 30% probability displacement ellipsoids. H atoms omitted for clarity.

**Fig. 2.**
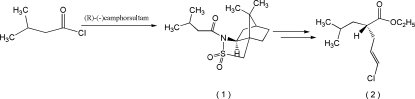
Preparation of the title compound.

## supplementary crystallographic information

### Comment

Aliskiren is a new renin inhibitor, useful for the treatment of hypertension and related cardiovascular diseases (Mariano *et al.*, 2008). In the process of synthesis of Aliskiren, the optically active compound ethyl (2*S*, 4E)-5-chloro-2-isopropylpent-4-enoate, (2 in Fig 2),  is a key intermediate. In order to obtain the compound (2), we used (*R*)-(-)camphorsultam as a chiral supplementary agent (Fig. 2). Thus, the title compound, (1), was prepared by reacting isovaleryl chloride with (*R*)-(-)camphorsultam.

The molecular structure of (1) is illustrated in Fig. 1. The five-membered ring (C4, C5, C10, N1, S1) shows nearly a C5β-envelope conformation, with the atom C5 deviating from the plane C4/C10/N1/S1 by 0.459 (4) Å. The plane formed by atom C2, C3, O3 and N1, have a dihedral angle of 19.6 (2)° with the aforementioned plane. In contrast, in the other independent molecule, the five-membered ring(C4', C5', C10', N1', S1') is close to a C10α-envelope conformation, with the atom C10 deviating from the plane C4'/C5'/N1'/S1' by 0.425 (4) Å. The plane (C2', C3', O3', N1') forms a dihedral angle of 23.9 (2)° with the aforementioned plane. Besides, there exist different bond lengths in the two independent molecules. The bond lengths of C1—C2 (1.508 Å), C1—C11 (1.524 Å) and C1—C12 (1.502 Å) are obviously longer than that of C1'-C2' (1.475 Å), C1'-C11' (1.508 Å) and C1'-C12' (1.476 Å), respectively.

In the crystal packing, there are two different intermolecular hydrogen bonds C—H···O (Table 1). The difference in the intermolecular hydrogen bonding interactions most possibly resulted in their slightly different bond lenghths and molecular conformations.

### Experimental

In a 250 ml bottomed flask was added camphorsultam(19 g, 88 mmol), toluene(100 ml), anhydrous triethylamine(16 ml, 110 mmol) and 4-dimethylaminopyridine(1.1 g, 10 mmol). The mixture was cooled to 273 K and then 12 ml of isovaleryl chloride were dripped in twenty minutes. After the addition, the mixture was stirred at 298 K until the TLC test showed that the reaction is complete. A solution of 13 ml of 36% hydrochloride acid in 19 ml of water was added at 298 K. The organic layers were separated and the aqueous layer was extracted for three times with 60 ml of dichloromethane. The combined organic layers were washed with water and concertrated to residue under vacuum, obtainning 26.7 g colourless crystal title compound.(yield 94%, m.p. 403–406 K). Since the crystal product was not found to be suitable for X-ray diffraction studies, a few crystals were dissolved in toluene, which was allowed to evaporate slowly to give colourless crystals of (1) suitable for X-ray diffraction studies.

### Refinement

All H atoms were placed in calculated positions (C—H 0.96–0.98 Å) and refined as riding with *U*_iso_(H) = 1.2–1.5Ueq of the parent atom.

### Crystal data

**Table:**

C_15_H_25_NO_3_S	*D*_x_ = 1.268 Mg m^−^^3^
*M**_r_* = 299.42	Mo *K*α radiation, λ = 0.71073 Å
Tetragonal, *P*4_1_	Cell parameters from 25 reflections
Hall symbol: P 4w	θ = 12–18°
*a* = 7.9038 (7) Å	µ = 0.21 mm^−^^1^
*c* = 50.228 (8) Å	*T* = 293 K
*V* = 3137.8 (7) Å^3^	Prism, colourless
*Z* = 8	0.30 × 0.30 × 0.20 mm
*F*(000) = 1296	

### Data collection

**Table:**

Bruker SMART CCD area-detector diffractometer	6416 independent reflections
Radiation source: fine-focus sealed tube	5184 reflections with *I* > 2σ(*I*)
graphite	*R*_int_ = 0.035
φ and ω scans	θ_max_ = 27.5°, θ_min_ = 2.6°
Absorption correction: multi-scan (*SADABS*; Sheldrick, 1996)	*h* = −10→5
*T*_min_ = 0.939, *T*_max_ = 0.959	*k* = −10→10
16320 measured reflections	*l* = −64→51

### Refinement

**Table:**

Refinement on *F*^2^	Hydrogen site location: inferred from neighbouring sites
Least-squares matrix: full	H-atom parameters constrained
*R*[*F*^2^ > 2σ(*F*^2^)] = 0.045	*w* = 1/[σ^2^(*F*_o_^2^) + (0.0521*P*)^2^ + 0.2775*P*] where *P* = (*F*_o_^2^ + 2*F*_c_^2^)/3
*wR*(*F*^2^) = 0.109	(Δ/σ)_max_ < 0.001
*S* = 1.04	Δρ_max_ = 0.38 e Å^−^^3^
6416 reflections	Δρ_min_ = −0.19 e Å^−^^3^
370 parameters	Extinction correction: *SHELXL97* (Sheldrick, 2008), Fc^*^=kFc[1+0.001xFc^2^λ^3^/sin(2θ)]^-1/4^
1 restraint	Extinction coefficient: 0.0164 (9)
Primary atom site location: structure-invariant direct methods	Absolute structure: Flack (1983), 2779 Friedel pairs
Secondary atom site location: difference Fourier map	Flack parameter: 0.03 (6)

### Special details

**Table:**

Geometry. All e.s.d.'s (except the e.s.d. in the dihedral angle between two l.s. planes) are estimated using the full covariance matrix. The cell e.s.d.'s are taken into account individually in the estimation of e.s.d.'s in distances, angles and torsion angles; correlations between e.s.d.'s in cell parameters are only used when they are defined by crystal symmetry. An approximate (isotropic) treatment of cell e.s.d.'s is used for estimating e.s.d.'s involving l.s. planes.
Refinement. Refinement of *F*^2^ against ALL reflections. The weighted *R*-factor *wR* and goodness of fit *S* are based on *F*^2^, conventional *R*-factors *R* are based on *F*, with *F* set to zero for negative *F*^2^. The threshold expression of *F*^2^ > σ(*F*^2^) is used only for calculating *R*-factors(gt) *etc*. and is not relevant to the choice of reflections for refinement. *R*-factors based on *F*^2^ are statistically about twice as large as those based on *F*, and *R*- factors based on ALL data will be even larger.

### Fractional atomic coordinates and isotropic or equivalent isotropic displacement parameters (Å^2^)

**Table:**

	*x*	*y*	*z*	*U*_iso_*/*U*_eq_	
S1	0.78736 (8)	0.43930 (9)	0.123672 (15)	0.04709 (19)	
N1	0.9609 (3)	0.3737 (3)	0.14051 (4)	0.0397 (5)	
O1	0.7275 (3)	0.5927 (3)	0.13478 (6)	0.0730 (7)	
O2	0.8219 (3)	0.4381 (3)	0.09586 (5)	0.0744 (7)	
O3	1.2414 (2)	0.3426 (3)	0.14096 (5)	0.0658 (6)	
C1	1.3005 (4)	0.6926 (4)	0.13051 (7)	0.0611 (9)	
H1	1.3948	0.6184	0.1264	0.073*	
C2	1.1411 (3)	0.6024 (4)	0.12208 (7)	0.0560 (8)	
H2A	1.0441	0.6718	0.1266	0.067*	
H2B	1.1422	0.5881	0.1029	0.067*	
C3	1.1225 (3)	0.4323 (4)	0.13503 (6)	0.0458 (7)	
C4	0.6596 (3)	0.2646 (4)	0.13288 (6)	0.0463 (7)	
H4A	0.5484	0.3030	0.1384	0.056*	
H4B	0.6464	0.1879	0.1179	0.056*	
C5	0.7480 (3)	0.1757 (3)	0.15582 (5)	0.0393 (6)	
C6	0.7286 (4)	−0.0169 (4)	0.15733 (7)	0.0569 (8)	
H6A	0.7891	−0.0725	0.1430	0.068*	
H6B	0.6105	−0.0501	0.1567	0.068*	
C7	0.8086 (5)	−0.0579 (4)	0.18471 (7)	0.0669 (10)	
H7A	0.7287	−0.1159	0.1962	0.080*	
H7B	0.9090	−0.1275	0.1827	0.080*	
C8	0.8533 (4)	0.1164 (4)	0.19581 (6)	0.0542 (7)	
H8	0.8647	0.1195	0.2152	0.065*	
C9	1.0083 (3)	0.1827 (4)	0.18085 (6)	0.0503 (7)	
H9A	1.1005	0.1018	0.1814	0.060*	
H9B	1.0466	0.2896	0.1881	0.060*	
C10	0.9399 (3)	0.2045 (3)	0.15241 (5)	0.0399 (6)	
H10	0.9879	0.1179	0.1407	0.048*	
C11	1.3250 (5)	0.8542 (6)	0.11444 (10)	0.0983 (16)	
H11A	1.2336	0.9308	0.1180	0.147*	
H11B	1.3266	0.8274	0.0958	0.147*	
H11C	1.4302	0.9062	0.1193	0.147*	
C12	1.3038 (6)	0.7248 (6)	0.15998 (8)	0.1006 (15)	
H12A	1.2079	0.7925	0.1649	0.151*	
H12B	1.4060	0.7835	0.1646	0.151*	
H12C	1.2996	0.6189	0.1693	0.151*	
C13	0.7110 (3)	0.2307 (4)	0.18487 (6)	0.0493 (7)	
C14	0.7325 (5)	0.4182 (4)	0.19173 (7)	0.0672 (9)	
H14A	0.7242	0.4330	0.2107	0.101*	
H14B	0.6455	0.4828	0.1831	0.101*	
H14C	0.8413	0.4564	0.1857	0.101*	
C15	0.5316 (4)	0.1818 (5)	0.19420 (7)	0.0732 (10)	
H15A	0.5200	0.2063	0.2128	0.110*	
H15B	0.5137	0.0631	0.1913	0.110*	
H15C	0.4494	0.2455	0.1843	0.110*	
S1'	0.90760 (10)	0.85718 (10)	0.063320 (15)	0.0537 (2)	
N1'	0.8697 (3)	1.0177 (3)	0.04197 (5)	0.0450 (5)	
O1'	1.0708 (3)	0.7888 (3)	0.05816 (6)	0.0780 (7)	
O2'	0.8725 (4)	0.9123 (3)	0.08967 (5)	0.0811 (7)	
O3'	0.8603 (3)	1.2988 (3)	0.03577 (5)	0.0767 (7)	
C1'	1.2148 (6)	1.3263 (6)	0.05161 (9)	0.0902 (13)	
H1'	1.1589	1.4337	0.0547	0.108*	
C2'	1.0962 (5)	1.1959 (5)	0.06127 (8)	0.0748 (10)	
H2'1	1.0676	1.2212	0.0796	0.090*	
H2'2	1.1537	1.0875	0.0611	0.090*	
C3'	0.9354 (4)	1.1795 (4)	0.04570 (7)	0.0557 (7)	
C4'	0.7438 (4)	0.7217 (4)	0.05125 (6)	0.0559 (8)	
H4'1	0.7895	0.6114	0.0468	0.067*	
H4'2	0.6569	0.7072	0.0647	0.067*	
C5'	0.6707 (4)	0.8062 (3)	0.02665 (6)	0.0479 (7)	
C6'	0.4805 (4)	0.7925 (4)	0.02243 (8)	0.0676 (9)	
H6'1	0.4192	0.8602	0.0353	0.081*	
H6'2	0.4429	0.6760	0.0237	0.081*	
C7'	0.4565 (5)	0.8626 (5)	−0.00620 (8)	0.0786 (11)	
H7'1	0.4012	0.7803	−0.0176	0.094*	
H7'2	0.3905	0.9661	−0.0061	0.094*	
C8'	0.6380 (5)	0.8958 (4)	−0.01530 (7)	0.0626 (9)	
H8'	0.6520	0.8977	−0.0347	0.075*	
C9'	0.7020 (4)	1.0534 (4)	−0.00152 (6)	0.0580 (8)	
H9'1	0.6254	1.1479	−0.0041	0.070*	
H9'2	0.8138	1.0845	−0.0078	0.070*	
C10'	0.7064 (3)	0.9970 (3)	0.02803 (6)	0.0454 (6)	
H10'	0.6164	1.0541	0.0380	0.054*	
C11'	1.3725 (5)	1.3300 (7)	0.06852 (10)	0.1022 (15)	
H11D	1.3431	1.3603	0.0864	0.153*	
H11E	1.4501	1.4119	0.0615	0.153*	
H11F	1.4245	1.2203	0.0684	0.153*	
C12'	1.2521 (7)	1.3223 (8)	0.02283 (10)	0.126 (2)	
H12D	1.3430	1.3991	0.0190	0.188*	
H12E	1.1532	1.3554	0.0130	0.188*	
H12F	1.2844	1.2098	0.0178	0.188*	
C13'	0.7403 (4)	0.7537 (4)	−0.00121 (6)	0.0556 (8)	
C14'	0.9302 (5)	0.7688 (5)	−0.00515 (8)	0.0767 (10)	
H14D	0.9861	0.6782	0.0040	0.115*	
H14E	0.9685	0.8752	0.0018	0.115*	
H14F	0.9560	0.7627	−0.0238	0.115*	
C15'	0.6873 (6)	0.5750 (4)	−0.00991 (8)	0.0832 (12)	
H15D	0.7228	0.5560	−0.0279	0.125*	
H15E	0.5666	0.5645	−0.0088	0.125*	
H15F	0.7396	0.4930	0.0015	0.125*	

### Atomic displacement parameters (Å^2^)

**Table:**

	*U*^11^	*U*^22^	*U*^33^	*U*^12^	*U*^13^	*U*^23^
S1	0.0360 (3)	0.0494 (4)	0.0558 (5)	0.0009 (3)	−0.0107 (3)	0.0148 (3)
N1	0.0341 (10)	0.0382 (11)	0.0469 (14)	0.0021 (8)	−0.0063 (9)	0.0072 (9)
O1	0.0484 (12)	0.0460 (12)	0.125 (2)	0.0105 (10)	−0.0056 (12)	0.0090 (12)
O2	0.0679 (14)	0.1078 (19)	0.0475 (15)	−0.0144 (13)	−0.0120 (11)	0.0301 (13)
O3	0.0346 (11)	0.0669 (14)	0.0960 (18)	0.0048 (9)	0.0010 (10)	0.0235 (12)
C1	0.0448 (16)	0.075 (2)	0.064 (2)	−0.0131 (14)	−0.0073 (14)	0.0119 (17)
C2	0.0432 (15)	0.0512 (16)	0.074 (2)	−0.0049 (12)	−0.0088 (15)	0.0164 (15)
C3	0.0339 (13)	0.0546 (16)	0.0490 (18)	0.0021 (11)	−0.0026 (12)	0.0072 (13)
C4	0.0357 (13)	0.0543 (16)	0.0490 (18)	−0.0043 (11)	−0.0108 (11)	0.0051 (13)
C5	0.0333 (12)	0.0438 (14)	0.0410 (16)	−0.0049 (10)	−0.0047 (10)	0.0004 (11)
C6	0.0621 (19)	0.0506 (17)	0.058 (2)	−0.0120 (14)	−0.0044 (15)	0.0027 (14)
C7	0.075 (2)	0.063 (2)	0.062 (2)	−0.0076 (16)	−0.0084 (18)	0.0244 (16)
C8	0.0567 (18)	0.069 (2)	0.0372 (17)	−0.0042 (14)	−0.0091 (13)	0.0158 (14)
C9	0.0427 (15)	0.0565 (17)	0.0517 (19)	0.0021 (12)	−0.0108 (13)	0.0132 (13)
C10	0.0367 (13)	0.0385 (13)	0.0446 (17)	0.0037 (10)	−0.0038 (11)	0.0055 (11)
C11	0.087 (3)	0.096 (3)	0.112 (4)	−0.048 (2)	−0.037 (2)	0.045 (3)
C12	0.130 (4)	0.111 (3)	0.061 (3)	−0.049 (3)	−0.010 (2)	−0.007 (2)
C13	0.0422 (15)	0.0645 (18)	0.0414 (18)	−0.0051 (13)	−0.0004 (12)	0.0017 (13)
C14	0.071 (2)	0.075 (2)	0.056 (2)	0.0015 (16)	0.0089 (17)	−0.0191 (17)
C15	0.0524 (19)	0.106 (3)	0.061 (2)	−0.0076 (18)	0.0063 (16)	0.008 (2)
S1'	0.0649 (5)	0.0533 (4)	0.0429 (4)	0.0029 (3)	−0.0098 (4)	0.0148 (3)
N1'	0.0519 (13)	0.0444 (13)	0.0387 (13)	0.0029 (10)	−0.0057 (10)	0.0107 (10)
O1'	0.0608 (14)	0.0726 (15)	0.101 (2)	0.0171 (12)	−0.0151 (13)	0.0223 (14)
O2'	0.126 (2)	0.0773 (16)	0.0398 (14)	−0.0129 (15)	−0.0058 (13)	0.0089 (12)
O3'	0.112 (2)	0.0461 (12)	0.0723 (17)	0.0019 (12)	−0.0297 (14)	0.0058 (11)
C1'	0.090 (3)	0.103 (3)	0.077 (3)	−0.022 (2)	−0.005 (2)	0.003 (2)
C2'	0.076 (2)	0.080 (2)	0.068 (2)	−0.0153 (19)	−0.0178 (19)	0.016 (2)
C3'	0.067 (2)	0.0531 (18)	0.0474 (19)	0.0002 (15)	−0.0044 (15)	0.0064 (14)
C4'	0.068 (2)	0.0507 (16)	0.049 (2)	−0.0011 (14)	−0.0020 (14)	0.0163 (14)
C5'	0.0565 (17)	0.0448 (15)	0.0424 (17)	0.0019 (12)	−0.0045 (13)	0.0096 (12)
C6'	0.067 (2)	0.067 (2)	0.069 (2)	−0.0115 (16)	−0.0078 (18)	0.0065 (17)
C7'	0.090 (3)	0.066 (2)	0.079 (3)	0.0008 (19)	−0.038 (2)	0.0051 (19)
C8'	0.094 (3)	0.0547 (18)	0.0389 (18)	0.0014 (16)	−0.0167 (16)	0.0085 (14)
C9'	0.075 (2)	0.0490 (16)	0.050 (2)	0.0049 (14)	−0.0130 (15)	0.0122 (14)
C10'	0.0495 (15)	0.0440 (14)	0.0426 (17)	0.0098 (11)	−0.0004 (12)	0.0035 (12)
C11'	0.071 (3)	0.133 (4)	0.103 (4)	−0.025 (2)	−0.009 (2)	−0.010 (3)
C12'	0.139 (4)	0.168 (5)	0.069 (3)	−0.072 (4)	0.025 (3)	−0.011 (3)
C13'	0.081 (2)	0.0451 (16)	0.0402 (18)	0.0032 (14)	−0.0020 (15)	0.0020 (12)
C14'	0.096 (3)	0.081 (2)	0.053 (2)	0.024 (2)	0.0205 (19)	−0.0012 (18)
C15'	0.128 (3)	0.0493 (19)	0.072 (3)	0.004 (2)	−0.010 (2)	−0.0041 (17)

### Geometric parameters (Å, °)

**Table:**

S1—O1	1.416 (2)	S1'—O2'	1.421 (3)
S1—O2	1.423 (2)	S1'—O1'	1.422 (2)
S1—N1	1.693 (2)	S1'—N1'	1.688 (2)
S1—C4	1.772 (3)	S1'—C4'	1.786 (3)
N1—C3	1.386 (3)	N1'—C3'	1.393 (4)
N1—C10	1.475 (3)	N1'—C10'	1.477 (3)
O3—C3	1.214 (3)	O3'—C3'	1.221 (4)
C1—C12	1.502 (5)	C1'—C2'	1.475 (5)
C1—C2	1.508 (4)	C1'—C12'	1.476 (6)
C1—C11	1.524 (5)	C1'—C11'	1.508 (6)
C1—H1	0.9700	C1'—H1'	0.9700
C2—C3	1.501 (4)	C2'—C3'	1.498 (5)
C2—H2A	0.9700	C2'—H2'1	0.9700
C2—H2B	0.9700	C2'—H2'2	0.9700
C4—C5	1.520 (4)	C4'—C5'	1.519 (4)
C4—H4A	0.9700	C4'—H4'1	0.9700
C4—H4B	0.9700	C4'—H4'2	0.9700
C5—C6	1.532 (4)	C5'—C6'	1.522 (4)
C5—C10	1.543 (3)	C5'—C10'	1.536 (4)
C5—C13	1.550 (4)	C5'—C13'	1.560 (4)
C6—C7	1.548 (5)	C6'—C7'	1.553 (5)
C6—H6A	0.9700	C6'—H6'1	0.9700
C6—H6B	0.9700	C6'—H6'2	0.9700
C7—C8	1.528 (5)	C7'—C8'	1.529 (5)
C7—H7A	0.9700	C7'—H7'1	0.9700
C7—H7B	0.9700	C7'—H7'2	0.9700
C8—C9	1.530 (4)	C8'—C9'	1.512 (4)
C8—C13	1.544 (4)	C8'—C13'	1.555 (4)
C8—H8	0.9800	C8'—H8'	0.9800
C9—C10	1.537 (4)	C9'—C10'	1.550 (4)
C9—H9A	0.9700	C9'—H9'1	0.9700
C9—H9B	0.9700	C9'—H9'2	0.9700
C10—H10	0.9800	C10'—H10'	0.9800
C11—H11A	0.9599	C11'—H11D	0.9599
C11—H11B	0.9599	C11'—H11E	0.9599
C11—H11C	0.9599	C11'—H11F	0.9599
C12—H12A	0.9599	C12'—H12D	0.9599
C12—H12B	0.9599	C12'—H12E	0.9599
C12—H12C	0.9599	C12'—H12F	0.9599
C13—C14	1.531 (4)	C13'—C14'	1.519 (5)
C13—C15	1.543 (4)	C13'—C15'	1.536 (4)
C14—H14A	0.9599	C14'—H14D	0.9599
C14—H14B	0.9599	C14'—H14E	0.9599
C14—H14C	0.9599	C14'—H14F	0.9599
C15—H15A	0.9599	C15'—H15D	0.9599
C15—H15B	0.9599	C15'—H15E	0.9599
C15—H15C	0.9599	C15'—H15F	0.9599
			
O1—S1—O2	117.15 (16)	O2'—S1'—O1'	117.62 (17)
O1—S1—N1	109.64 (13)	O2'—S1'—N1'	109.08 (14)
O2—S1—N1	109.45 (13)	O1'—S1'—N1'	109.31 (14)
O1—S1—C4	111.95 (15)	O2'—S1'—C4'	110.99 (16)
O2—S1—C4	111.11 (14)	O1'—S1'—C4'	111.57 (16)
N1—S1—C4	95.32 (11)	N1'—S1'—C4'	96.10 (12)
C3—N1—C10	119.2 (2)	C3'—N1'—C10'	119.4 (2)
C3—N1—S1	123.05 (18)	C3'—N1'—S1'	122.6 (2)
C10—N1—S1	112.89 (15)	C10'—N1'—S1'	111.92 (17)
C12—C1—C2	111.8 (3)	C2'—C1'—C12'	115.7 (4)
C12—C1—C11	112.2 (3)	C2'—C1'—C11'	110.7 (4)
C2—C1—C11	110.7 (3)	C12'—C1'—C11'	112.8 (4)
C12—C1—H1	107.3	C2'—C1'—H1'	105.6
C2—C1—H1	107.3	C12'—C1'—H1'	105.6
C11—C1—H1	107.3	C11'—C1'—H1'	105.6
C3—C2—C1	112.6 (2)	C1'—C2'—C3'	115.3 (3)
C3—C2—H2A	109.1	C1'—C2'—H2'1	108.4
C1—C2—H2A	109.1	C3'—C2'—H2'1	108.4
C3—C2—H2B	109.1	C1'—C2'—H2'2	108.4
C1—C2—H2B	109.1	C3'—C2'—H2'2	108.4
H2A—C2—H2B	107.8	H2'1—C2'—H2'2	107.5
O3—C3—N1	118.0 (2)	O3'—C3'—N1'	118.2 (3)
O3—C3—C2	123.6 (2)	O3'—C3'—C2'	124.0 (3)
N1—C3—C2	118.4 (2)	N1'—C3'—C2'	117.8 (3)
C5—C4—S1	107.21 (17)	C5'—C4'—S1'	106.76 (19)
C5—C4—H4A	110.3	C5'—C4'—H4'1	110.4
S1—C4—H4A	110.3	S1'—C4'—H4'1	110.4
C5—C4—H4B	110.3	C5'—C4'—H4'2	110.4
S1—C4—H4B	110.3	S1'—C4'—H4'2	110.4
H4A—C4—H4B	108.5	H4'1—C4'—H4'2	108.6
C4—C5—C6	116.8 (2)	C4'—C5'—C6'	117.3 (2)
C4—C5—C10	107.4 (2)	C4'—C5'—C10'	109.0 (2)
C6—C5—C10	104.5 (2)	C6'—C5'—C10'	104.9 (2)
C4—C5—C13	119.8 (2)	C4'—C5'—C13'	118.6 (2)
C6—C5—C13	102.3 (2)	C6'—C5'—C13'	101.8 (3)
C10—C5—C13	104.4 (2)	C10'—C5'—C13'	103.7 (2)
C5—C6—C7	102.2 (2)	C5'—C6'—C7'	103.0 (3)
C5—C6—H6A	111.3	C5'—C6'—H6'1	111.2
C7—C6—H6A	111.3	C7'—C6'—H6'1	111.2
C5—C6—H6B	111.3	C5'—C6'—H6'2	111.2
C7—C6—H6B	111.3	C7'—C6'—H6'2	111.2
H6A—C6—H6B	109.2	H6'1—C6'—H6'2	109.1
C8—C7—C6	103.3 (2)	C8'—C7'—C6'	102.9 (3)
C8—C7—H7A	111.1	C8'—C7'—H7'1	111.2
C6—C7—H7A	111.1	C6'—C7'—H7'1	111.2
C8—C7—H7B	111.1	C8'—C7'—H7'2	111.2
C6—C7—H7B	111.1	C6'—C7'—H7'2	111.2
H7A—C7—H7B	109.1	H7'1—C7'—H7'2	109.1
C7—C8—C9	108.3 (3)	C9'—C8'—C7'	108.6 (3)
C7—C8—C13	103.3 (2)	C9'—C8'—C13'	102.3 (2)
C9—C8—C13	102.0 (2)	C7'—C8'—C13'	103.2 (3)
C7—C8—H8	114.0	C9'—C8'—H8'	113.9
C9—C8—H8	114.0	C7'—C8'—H8'	113.9
C13—C8—H8	114.0	C13'—C8'—H8'	113.9
C8—C9—C10	102.3 (2)	C8'—C9'—C10'	102.1 (2)
C8—C9—H9A	111.3	C8'—C9'—H9'1	111.4
C10—C9—H9A	111.3	C10'—C9'—H9'1	111.4
C8—C9—H9B	111.3	C8'—C9'—H9'2	111.4
C10—C9—H9B	111.3	C10'—C9'—H9'2	111.4
H9A—C9—H9B	109.2	H9'1—C9'—H9'2	109.2
N1—C10—C9	116.0 (2)	N1'—C10'—C5'	106.9 (2)
N1—C10—C5	106.83 (19)	N1'—C10'—C9'	116.2 (2)
C9—C10—C5	103.1 (2)	C5'—C10'—C9'	103.6 (2)
N1—C10—H10	110.2	N1'—C10'—H10'	109.9
C9—C10—H10	110.2	C5'—C10'—H10'	109.9
C5—C10—H10	110.2	C9'—C10'—H10'	109.9
C1—C11—H11A	109.5	C1'—C11'—H11D	109.5
C1—C11—H11B	109.5	C1'—C11'—H11E	109.5
H11A—C11—H11B	109.5	H11D—C11'—H11E	109.5
C1—C11—H11C	109.5	C1'—C11'—H11F	109.5
H11A—C11—H11C	109.5	H11D—C11'—H11F	109.5
H11B—C11—H11C	109.5	H11E—C11'—H11F	109.5
C1—C12—H12A	109.5	C1'—C12'—H12D	109.5
C1—C12—H12B	109.5	C1'—C12'—H12E	109.5
H12A—C12—H12B	109.5	H12D—C12'—H12E	109.5
C1—C12—H12C	109.5	C1'—C12'—H12F	109.5
H12A—C12—H12C	109.5	H12D—C12'—H12F	109.5
H12B—C12—H12C	109.5	H12E—C12'—H12F	109.5
C14—C13—C15	106.0 (3)	C14'—C13'—C15'	107.7 (3)
C14—C13—C8	113.9 (2)	C14'—C13'—C8'	113.4 (3)
C15—C13—C8	114.5 (3)	C15'—C13'—C8'	113.1 (3)
C14—C13—C5	117.5 (2)	C14'—C13'—C5'	116.4 (3)
C15—C13—C5	112.9 (2)	C15'—C13'—C5'	113.8 (3)
C8—C13—C5	91.9 (2)	C8'—C13'—C5'	91.9 (2)
C13—C14—H14A	109.5	C13'—C14'—H14D	109.5
C13—C14—H14B	109.5	C13'—C14'—H14E	109.5
H14A—C14—H14B	109.5	H14D—C14'—H14E	109.5
C13—C14—H14C	109.5	C13'—C14'—H14F	109.5
H14A—C14—H14C	109.5	H14D—C14'—H14F	109.5
H14B—C14—H14C	109.5	H14E—C14'—H14F	109.5
C13—C15—H15A	109.5	C13'—C15'—H15D	109.5
C13—C15—H15B	109.5	C13'—C15'—H15E	109.5
H15A—C15—H15B	109.5	H15D—C15'—H15E	109.5
C13—C15—H15C	109.5	C13'—C15'—H15F	109.5
H15A—C15—H15C	109.5	H15D—C15'—H15F	109.5
H15B—C15—H15C	109.5	H15E—C15'—H15F	109.5
			
O1—S1—N1—C3	−84.0 (2)	O2'—S1'—N1'—C3'	50.2 (3)
O2—S1—N1—C3	45.8 (3)	O1'—S1'—N1'—C3'	−79.7 (3)
C4—S1—N1—C3	160.5 (2)	C4'—S1'—N1'—C3'	164.9 (3)
O1—S1—N1—C10	121.1 (2)	O2'—S1'—N1'—C10'	−102.0 (2)
O2—S1—N1—C10	−109.1 (2)	O1'—S1'—N1'—C10'	128.1 (2)
C4—S1—N1—C10	5.5 (2)	C4'—S1'—N1'—C10'	12.7 (2)
C12—C1—C2—C3	−61.0 (4)	C12'—C1'—C2'—C3'	−49.4 (6)
C11—C1—C2—C3	173.1 (3)	C11'—C1'—C2'—C3'	−179.3 (4)
C10—N1—C3—O3	−1.8 (4)	C10'—N1'—C3'—O3'	−4.5 (4)
S1—N1—C3—O3	−155.3 (2)	S1'—N1'—C3'—O3'	−154.8 (3)
C10—N1—C3—C2	178.0 (3)	C10'—N1'—C3'—C2'	176.6 (3)
S1—N1—C3—C2	24.5 (4)	S1'—N1'—C3'—C2'	26.4 (4)
C1—C2—C3—O3	−32.1 (5)	C1'—C2'—C3'—O3'	−36.2 (6)
C1—C2—C3—N1	148.1 (3)	C1'—C2'—C3'—N1'	142.5 (4)
O1—S1—C4—C5	−99.1 (2)	O2'—S1'—C4'—C5'	119.9 (2)
O2—S1—C4—C5	127.8 (2)	O1'—S1'—C4'—C5'	−106.8 (2)
N1—S1—C4—C5	14.6 (2)	N1'—S1'—C4'—C5'	6.7 (2)
S1—C4—C5—C6	−146.5 (2)	S1'—C4'—C5'—C6'	−142.3 (3)
S1—C4—C5—C10	−29.6 (3)	S1'—C4'—C5'—C10'	−23.4 (3)
S1—C4—C5—C13	89.1 (2)	S1'—C4'—C5'—C13'	94.8 (3)
C4—C5—C6—C7	−171.6 (3)	C4'—C5'—C6'—C7'	−170.4 (3)
C10—C5—C6—C7	69.9 (3)	C10'—C5'—C6'—C7'	68.6 (3)
C13—C5—C6—C7	−38.7 (3)	C13'—C5'—C6'—C7'	−39.3 (3)
C5—C6—C7—C8	3.2 (3)	C5'—C6'—C7'—C8'	3.8 (3)
C6—C7—C8—C9	−74.1 (3)	C6'—C7'—C8'—C9'	−74.8 (3)
C6—C7—C8—C13	33.6 (3)	C6'—C7'—C8'—C13'	33.2 (3)
C7—C8—C9—C10	65.4 (3)	C7'—C8'—C9'—C10'	65.7 (3)
C13—C8—C9—C10	−43.2 (3)	C13'—C8'—C9'—C10'	−42.9 (3)
C3—N1—C10—C9	66.2 (3)	C3'—N1'—C10'—C5'	179.0 (3)
S1—N1—C10—C9	−137.8 (2)	S1'—N1'—C10'—C5'	−27.8 (3)
C3—N1—C10—C5	−179.5 (2)	C3'—N1'—C10'—C9'	63.9 (3)
S1—N1—C10—C5	−23.5 (3)	S1'—N1'—C10'—C9'	−142.9 (2)
C8—C9—C10—N1	125.2 (2)	C4'—C5'—C10'—N1'	32.4 (3)
C8—C9—C10—C5	8.9 (3)	C6'—C5'—C10'—N1'	158.8 (2)
C4—C5—C10—N1	33.5 (3)	C13'—C5'—C10'—N1'	−94.8 (3)
C6—C5—C10—N1	158.2 (2)	C4'—C5'—C10'—C9'	155.7 (2)
C13—C5—C10—N1	−94.7 (2)	C6'—C5'—C10'—C9'	−78.0 (3)
C4—C5—C10—C9	156.2 (2)	C13'—C5'—C10'—C9'	28.5 (3)
C6—C5—C10—C9	−79.1 (3)	C8'—C9'—C10'—N1'	125.4 (3)
C13—C5—C10—C9	28.0 (3)	C8'—C9'—C10'—C5'	8.5 (3)
C7—C8—C13—C14	−176.2 (3)	C9'—C8'—C13'—C14'	−62.0 (4)
C9—C8—C13—C14	−63.8 (3)	C7'—C8'—C13'—C14'	−174.7 (3)
C7—C8—C13—C15	61.5 (3)	C9'—C8'—C13'—C15'	174.9 (3)
C9—C8—C13—C15	173.9 (3)	C7'—C8'—C13'—C15'	62.2 (4)
C7—C8—C13—C5	−54.8 (3)	C9'—C8'—C13'—C5'	57.9 (3)
C9—C8—C13—C5	57.6 (2)	C7'—C8'—C13'—C5'	−54.8 (3)
C4—C5—C13—C14	−53.7 (3)	C4'—C5'—C13'—C14'	−55.3 (4)
C6—C5—C13—C14	175.3 (3)	C6'—C5'—C13'—C14'	174.4 (3)
C10—C5—C13—C14	66.5 (3)	C10'—C5'—C13'—C14'	65.6 (3)
C4—C5—C13—C15	70.3 (3)	C4'—C5'—C13'—C15'	71.0 (4)
C6—C5—C13—C15	−60.8 (3)	C6'—C5'—C13'—C15'	−59.3 (3)
C10—C5—C13—C15	−169.5 (3)	C10'—C5'—C13'—C15'	−168.1 (3)
C4—C5—C13—C8	−172.1 (2)	C4'—C5'—C13'—C8'	−172.7 (3)
C6—C5—C13—C8	56.9 (2)	C6'—C5'—C13'—C8'	57.0 (3)
C10—C5—C13—C8	−51.8 (2)	C10'—C5'—C13'—C8'	−51.7 (3)

### Hydrogen-bond geometry (Å, °)

**Table:**

*D*—H···*A*	*D*—H	H···*A*	*D*···*A*	*D*—H···*A*
C4—H4A···O3^i^	0.97	2.45	3.387 (3)	162
C15'—H15F···O3'^ii^	0.96	2.49	3.450 (5)	173

Symmetry codes: (i) *x*−1, *y*, *z*; (ii) *x*, *y*−1, *z*.
